# Ion Channels in Brain Metastasis

**DOI:** 10.3390/ijms17091513

**Published:** 2016-09-08

**Authors:** Lukas Klumpp, Efe C. Sezgin, Franziska Eckert, Stephan M. Huber

**Affiliations:** 1 Department of Radiation Oncology, University of Tübingen, 72076 Tübingen, Germany; lukas.klumpp@med.uni-tuebingen.de (L.K.); efe.sezgin@uni-tuebingen.de (E.C.S.); franziska.eckert@med.uni-tuebingen.de (F.E.); 2 Dr. Margarete Fischer-Bosch-Institute of Clinical Pharmacology, 70376 Stuttgart, Germany

**Keywords:** voltage-gated sodium channels, pannexin, connexin, K_v_10.1, BK_Ca_

## Abstract

Breast cancer, lung cancer and melanoma exhibit a high metastatic tropism to the brain. Development of brain metastases severely worsens the prognosis of cancer patients and constrains curative treatment options. Metastasizing to the brain by cancer cells can be dissected in consecutive processes including epithelial–mesenchymal transition, evasion from the primary tumor, intravasation and circulation in the blood, extravasation across the blood–brain barrier, formation of metastatic niches, and colonization in the brain. Ion channels have been demonstrated to be aberrantly expressed in tumor cells where they regulate neoplastic transformation, malignant progression or therapy resistance. Moreover, many ion channel modulators are FDA-approved drugs and in clinical use proposing ion channels as druggable targets for future anti-cancer therapy. The present review article aims to summarize the current knowledge on the function of ion channels in the different processes of brain metastasis. The data suggest that certain channel types involving voltage-gated sodium channels, ATP-release channels, ionotropic neurotransmitter receptors and gap junction-generating connexins interfere with distinct processes of brain metastazation.

## 1. Introduction

Brain metastasis represents the most frequent form of brain malignancy. Primary tumors that frequently disseminate to the brain are lung and breast cancers as well as melanoma. A significant fraction (30%) of all patients with systemic cancer develops brain metastases. At the time of first diagnosis, half of the patients with brain metastasis present with multiple brain lesions [1]. Brain metastases similar to primary brain tumors impair brain function by dislodging or killing neurons and inducing cerebral edema, which result in a decline of neurocognitive performance and intracranial pressure, a major cause of death in patients with brain malignancies. Patients with brain metastasis have a very poor prognosis with a median survival time in the range of few weeks from the time of diagnosis. With multimodal therapy regimes that may combine surgical resection or stereotactic radiosurgery with whole brain irradiation and/or systemic therapy, median survival time can be extended to few months up to 1.5 years (for review see [2]).

Mestastasizing to the brain involves several processes. Tumor cells have to adopt a migratory phenotype to evade from the primary tumor and to transmigrate through the endothelial barrier into vessels in order to reach the blood circulation. Circulating tumor cells floating in the blood stream have to survive the mechanical forces, to express surface molecules that address them to the brain, to specifically bind to the endothelium of brain blood vessels, and to transmigrate the blood–brain barrier. Upon extravasation in the brain, tumor cells have to defend the stress signals of neural stem cells and activated microglia and astrocytes. This often results in dormancy of the tumor cells before restarting colonization of the brain parenchyma. A prerequisite for colonization is the formation of a metastatic niche by a close association of the tumor cells with the brain endothelium stimulating angiogenesis. Moreover, tumor cells reciprocally interact with microglia and astrocytes harnessing their pro-tumoral and survival-promoting functions, and recruit bone marrow-derived myeloid cells which accomplish vasculogenesis by transition into endothelial cells (for review see [2]).

Furthermore, tumor cells either pre-express or adopt a neuronal phenotype in the brain by transcriptional upregulation of genes normally expressed in the cells of the neuronal lineage. This neuro-mimicry improves the adaptation to the brain microenvironment which may boost survival and colonization in the brain and outgrowth as brain metastases (for review see [2]). Expansion of brain metastases may occur with clearly delineated borders. The majority of metastases (50%–70%), however, forms satellites in, diffusively infiltrates, protrudes perivascularly, or infiltrates the adjacent normal brain tissue in an angio-cooptive manner [3] impeding complete neurosurgical resection. Finally, tumor cells within the brain microenvironment may acquire therapy resistance during treatment that further may decrease the efficacy of radio- and/or chemotherapy [4,5]. In summary, the progression of a curable primary tumor to an often palliative therapy situation by metastasizing to the brain is a sequence of multiple steps that rely on the cell–cell interactions in three different (micro)environments (primary tumor, blood, and brain) and cell physiological processes such as cell migration and intra- and extravasation.

Tumor cells are endowed with an ion channel toolkit that differs considerably from that of the parental non-transformed cells. Certain types of ion channels seem to have a high oncogenic potential and are preferentially over-expressed in tumors. These “oncochannels” in plasma or intracellular membranes reportedly exert pivotal functions in tumor biology by promoting neoplastic transformation, malignant progression, metastasis, and therapy resistance (for review see [6,7,8]). Importantly, besides executing cell physiological functions, ion channels as integral modules crosstalk with biochemical signaling thereby regulating virtually all aspects of cell biology. For instance, human ether-à-go-go-related (hERG) K^+^ channels in neuroblastoma cells are physically linked to β1 integrins and transduce cell adhesion-dependent signaling to downstream effector proteins such as the focal adhesion kinase [9]. Another intriguing example is given by the interference of the “oncochannel” ether-à-go-go-1 (EAG1) with the aberrant signaling in tumor cells: EAG1 is highly overexpressed in many tumor entities and sole transfection of Chinese hamster ovary cells with EAG1 (but not with a less oncogenic voltage gated K_v_ K^+^ channel type used for control) is sufficient to render CHO cells highly tumorigenic and malignant progressing when grown ectopically in immunocompromised mice [10].

The prevalence of brain metastasis increases. This is probably in part due to better neuroimaging techniques. A second reason is the more effective systemic treatment of the primary disease and extracranial metastases. Novel systemic therapies include large molecules such as trastuzumab which are hardly able to permeate the blood–brain barrier [11]. Thus, novel approaches for treatment concepts aiming for cure or at least local control that can be offered to patients with brain metastasis are urgently needed [12].

Ion channel targeting might be such an approach for two reasons: the exclusive overexpression of certain ion channel variants by tumor cells renders ion channel targeting tumor-selective. Secondly, many drugs with on-target or off-target ion channel-modulating function are in clinical use. Among the former are pharmaceuticals such as verapamil, pregabalin, sotalol, fampridine, ziconotide, lidocaine, lamotrigine, riluzole, amiloride or ivacaftor used to target voltage-gated Ca^2+^-, K^+^- or Na^+^ channels or epithelial Na^+^- or Cl^−^ channels for the treatment of hypertension, pain, arrhythmia, multiple sclerosis, epilepsy, amyotrophic lateral sclerosis, hypokalaemia, or cystic fibrosis [13,14,15,16]. Moreover, a great number of specific ion channel modulators have been developed or are already tested in clinical trials [14,15,17]). As a proof of concept, several preclinical in vivo studies mainly on primary tumors could indeed demonstrate significant anti-tumor effects of ion channel-targeting drugs (for review see [18]). Combined, this suggests that potentially brain metastasis-promoting ion channels might be promising and druggable new targets for the treatment of brain metastasis.

The present review article, therefore, aims to summarize the few data on ion channel function in the different processes of brain metastasis. In addition, it discusses clinical data from cancer patients with brain metastases receiving drugs with ion channel-modulating action. To begin with, ion channel types that promote metastasis will be introduced.

## 2. Epithelial–Mesenchymal Transition

**Figure 1:**
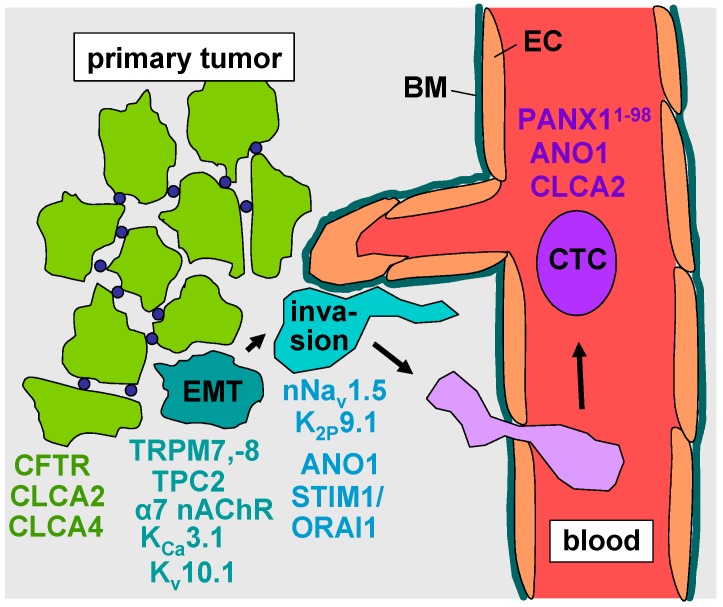
Synopsis of ion channel types involved in evasion of cancer cells from the primary tumor. EMT: epithelial–mesenchymal transition; CTC: circulating cancer cells; EC: endothelial cell; BM: basement membrane. Arrows define the sequence of processes leading to intravasation of the tumor cells.

As initial step of metastasis, cells have to adopt a migratory and invasive phenotype by epithelial–mesenchymal transition (EMT), to detach and emigrate from the primary tumor. Intracellular and plasma membrane ion channels have been demonstrated to regulate EMT in tumors with high metastatic brain tropism such as lung or breast cancer. In breast cancer cell lines, EMT is associated with remodeling of the Ca^2+^ signalosome [19,20,21] and partly depends on the activity of member 7 (TRPM7) and 8 (TRPM8) of the melastatin family of the transient receptor potential nonselective cation channel superfamily. Knock-down experiments disclosed STAT3 and the Akt/Glycogen synthase kinase 3 beta (GSK-3β) pathway as downstream targets of TRPM7- [22] and TRPM8- [23] mediated Ca^2+^-signaling, respectively. In addition, the activity of the Ca^2+^-permeable two pore segment channel-2 (TPC2) in the membrane of endolysomal Ca^2+^ stores is reportedly required for Epithelial Growth Factor (EGF)-induced EMT [24]. The alpha-7 nicotinic receptor (α7 nAChR) is a further Ca^2+^-permeable cation channel stimulating EMT and a migratory invasive phenotype in breast and lung cancer cell lines [25]. Moreover, transforming growth factor beta-1 (TGFβ1)-induced EMT is paralleled by upregulation of voltage-gated EAG1 (Ether-à-go-go-1, K_v_10.1) K^+^ channels in lung adenocarcinoma cells [26] and inhibited by K_Ca_3.1 (IK) K^+^ channel blockade in bronchial epithelial cells [27]. This might hint to and prove an EMT-promoting function of EAG1 and K_Ca_3.1, respectively (Figure 1, turquoise).

Unlike these cation channels, the Cl^−^ channel CFTR (cystic fibrosis transmembrane conductance regulator) and the Ca^2+^-activated Cl^−^ channel regulators CLCA2 [28,29] and CLCA4 [30] (chloride channel accessory protein-2, -4 [31,32]) suppress EMT in breast cancer cells possibly by counteracting oncogenic alkalinization of intacellular pH. In addition, CLCA2 has been shown to be a p53 target gene that transcriptionally inactivates focal-adhesion kinase (FAK) and thereby inhibits invasion [33]. CLCA2 is silenced by DNA methylation in breast cancers [34]. Experimental knockdown of CFTR, CLCA2 or CLCA4 mimics TGFβ1-induced EMT. Accordingly, patients with low CFTR, CLCA2 or CLCA4 mRNA abundance in breast cancer specimens have higher odds of developing metastases and a decreased relapse-free survival [29,30,35] (Figure 1, green). Combined, these data suggest complex regulation of EMT in breast and lung cancer by several ion channel types. Besides controlling EMT, ion channels have been demonstrated to be key drivers of cancer cell invasion.

## 3. Evasion from the Primary Tumor

Voltage-gated Na^+^ channels (VGSCs) have been identified in several primary tumor entities including breast cancer, lung cancer and melanoma to be upregulated and to promote tissue invasion (for review see [36,37]). VGSCs consist of one pore-forming α subunit (Na_v_1.1–Na_v_1.9) and one or more regulatory β subunits (β1–β4). The latter modulate channel gating and, in addition, exert cell adhesion function via their extracellular immunoglobulin loop [38,39]. VGSCs generate action potentials in excitable cells such as neurons, skeletal and cardiac myocytes. They are also expressed in non-excitable cells including fibroblasts, immune cells, glia, and several tumor entities. Moreover, VGSCs regulate cell proliferation, neurite outgrowth and neuronal pathfinding and migration during neurogenesis of the central nervous system. Upregulation of Na_v_1.5, Na_v_1.6, and/or Na_v_1.7 α-subunits has been described in melanoma, lung and breast cancer (for review see [36]). In particular, in breast cancer, a neonatal splice variant of Na_v_1.5 has been identified [40,41] hinting to re-expression of ontogenetic programs by the cancer cells. High abundance of Na_v_1.5-encoding mRNA in breast cancer specimens seems to be associated with an increased risk of lymph node metastasis [40] and tumor recurrence, as well as with lower overall survival [42]. Accordingly, Na_v_1.5 knock-down [43] or the antiepileptic drug phenytoin [44] and the antianginal drug ranolazine [45], both Na_v_1.5 inhibitors, attenuate breast cancer metastasis in an orthotopic breast cancer and a tail vein cancer cell injection mouse model, respectively. Likewise, inhibiting VGSCs with local injections of tetrodotoxin blunted metastasis in the Copenhagen rat model of prostate cancer [46] (Figure 1, blue).

Since cancer cells usually exhibit lower plasma membrane potentials than the parental normal cells, persistent VGSC-generated Na^+^ currents (i.e., the Na^+^ currents that do not inactivate during depolarization of the plasma membrane) have been suggested to couple the activity of VGSCs in cancer cells to downstream signaling pathways ([38], for review see [36]). Mechanistically, VGSCs have been located in the cytoplasms, in particular in the perinuclear region, and in the lamellipodium of highly metastatic cancer cells [42,47]. Moreover, they are in complex with the Na^+^/H^+^ antiporter (NHE1) and activate NHE1 by yet unknown mechanisms. NHE1-mediated proton extrusion, in turn, leads to intracellular alkalinization and an extracellular peri-membrane acidification, which subsequently promotes invasion by increasing the activity of cysteine cathepsins and the digestion of the extracellular matrix by these acidic proteases [48,49].

Beyond mediating pH signaling, VGSCs have been reported to interfere with Ca^2+^ signaling. Depolarizing Na^+^ influx may activate voltage-gated Ca^2+^ channels in the plasma membrane. In addition, local Na^+^-overload may reverse the working mode of the plasma membrane Na^+^/Ca^2+^ exchanger (for review see [36]) resulting in Na^+^-overload-mediated Ca^2+^ uptake. Moreover, activation of VGSCs in intracellular Na^+^ store vesicles releases Na^+^ into the cytosol that is buffered by subsequent Na^+^ uptake into the mitochondria by Na^+^/Ca^2+^ exchange resulting in Ca^2+^-liberation from the mitochondria [50].

In this context, store-operated Ca^2+^ influx via STIM1/ORAI1 has been shown to regulate invadopodia formation in lung cancer [51] and to be pivotal in breast cancer cells for exteriorization of cell surface-associated enolase-1 (ENO-1) that enhances plasmin formation and hence pericellular proteolysis [52]. Along those lines, K_2P_9.1, a two-pore domain potassium (K2P) channel, encoded by the *KCNK9* gene, is overexpressed in lung and breast cancer. K_2P_9.1 contributes to formation and maintenance of the membrane potential which is required for continued store-operated Ca^2+^ influx. In breast and lung cancer patients, elevated *KCNK9* mRNA abundance associates with shorter patient survival. In the tail vein, cancer cell injection mouse model, systemic application of a K_2P_9.1-directed antibody which binds extracellularly and induces channel internalization inhibits murine breast cancer metastases [53]. Moreover, the Ca^2+^-activated Cl^−^ channel ANO1 (anoctamin-1, transmembrane member 16A, TMEM16A) is overexpressed in lung cancer cells where it promotes invasion [54]. In combination, these data suggest a key function of VGSC- and K_2P_9.1-regulated Ca^2+^ signals in cancer cell invasion (Figure 1, blue).

Finally, VGSCs have been demonstrated in colon carcinoma to be a key regulator of the transcriptional network that promotes cancer cell invasion [55]. In breast cancer cells, upregulation of downstream targets of Na_v_1.5 such as matrix metalloproteinases rely on Na_v_1.5 conductive function [47]. Notably, neonatal Na_v_1.5 activity upregulates its own mRNA and protein expression in a positive feedback loop in metastatic breast cancer [56]. Collectively, these observations indicate that ion channels such as neonatal Na_v_1.5 regulate tumor cell invasion in a highly complex manner.

## 4. Intravasation, Circulation, Brain Tropism, and Transmigration of the Blood–Brain Barrier

Brain metastases are most probably initiated in the primary tumor by direct intravasation into blood and not via lymph vessels. Intravasation may be facilitated by tumor-induced modulation of the vasculature including angiogenetic formation of immature tumor vasculature, dilatation of vessels and permeabilization of the endothelial barrier as well as by reprogrammed co-evading assistance cells such as macrophages. Intravasation may occur via multicellular streams, shedding of endothelium-lined outpocketing of the tumor into a sinusoidal space, transcellular and paracellular diapedesis by single cells, or vascular mimicry of tumor cells which form part of the endothelial layer before becoming released into the blood (for review see [57]). One may assume that processes such as diapedesis with tumor cells having to squeeze through narrow paracellular or trancellular routes requires pronounced cell volume changes which critically depend on ion channel activities. The nature of these channels, however, is not defined since data on the contribution of ion channels in intravasation of tumor cells—to the best of our knowledge—have not been published. Upon entering the vessels, tumor cells circulate in the blood stream. The next paragraphs introduce ion channels found in circulating tumor cells (CTCs).

As described earlier, the CLCA2 Cl^−^ channel regulator exerts tumor-suppressing function in breast cancer. In lung cancer-derived CTCs, in contrast, CLCA2 is upregulated and can be used as CTC marker [58,59]. Similarly, anoctamin-1 (ANO1, TMEM16A), a Ca^2+^-activated Cl^−^ channel serves as tumor marker in gastrointestinal stromal tumor-derived CTCs. Analysis of patient data revealed a prognostic and predictive value of CLCA2 and ANO1 mRNA abundance in CTCs for lung [59] and gastrointestinal [60] cancer, respectively. The functional significance of these channels/regulators for the survival of CTCs has not been defined yet (Figure 1, violet).

To reach the brain, CTCs (except for lung cancer) have to travel at least once through the capillary bed of the lung. In doing so, CTCs are exposed to strong mechanical shear stress and deformation forces generated by the blood flow and the narrow lumina of the microvessels. CTCs are deformed from a spherical towards an elongated, cylindrical morphology when passing the microvasculature. This deformation has been demonstrated to induce mechanical traumata [61] which trigger apoptotic cell death in the vast majority of CTCs leading to so-called metastatic inefficiency [62]. Since ANO1 has been implicated in local cell volume decrease of cancer cells by generating Cl^−^ efflux that accompanies K^+^ efflux which together drive osmotically obliged H_2_O efflux [63] it is tempting to speculate that ANO1 and maybe also CLCA2 function might prevent cell death during mechanical deformation of CTCs.

Whole-transcriptome RNA sequencing of in vivo-selected highly metastatic sub-lines of human breast cancer cells revealed an activating mutation in the ATP release channel pannexin-1 (PANX1^1−98^) [64]. PANX1^1−98^ functions in the plasma membrane of breast cancer-derived CTCs as a mechanosensitive channel that generates a cell deformation-triggered efflux of ATP into the microvessel lumen. Extracellular ATP in turn stimulates metabotropic P2Y purinoceptors of the CTCs in an autocrine manner which inhibits apoptosis and contributes to the survival of CTCs and increases metastasis efficiency [64] (Figure 1, violet).

A contribution of ion channels to the brain tropisms of, e.g., circulating lung cancer cells—to our best knowledge—has not been reported. Due to the absence of lymph vessels in the brain, CTCs have to bind to endothelium of blood vessels and to break through the blood brain barrier (BBB) in order to home, colonize and outgrow in the brain parenchyma. The BBB is composed of a unfenestrated, tight endothelium surrounded by a basement membrane, ensheathed by pericytes, smooth muscle cells (except in capillaries) and end-feet of astrocytes that form a perivascular sheath and restrict water fluxes, paracellular ion fluxes and the permeability of macromolecules.

**Figure 2:**
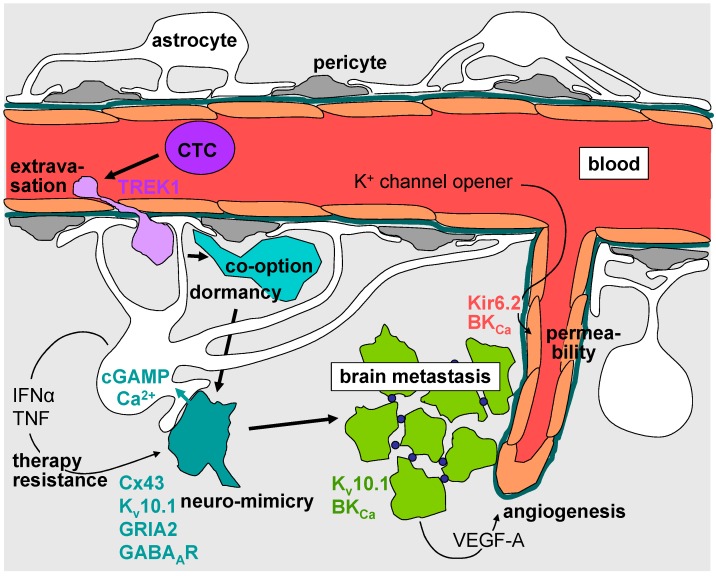
Synopsis of ion channel types involved in extravasation of cancer cells in the brain, neuromimicry, and modulation of the permeability of the brain blood cancer barrier. CTC: circulating cancer cells; VEGF-A: vascular endothelial growth factor A; IFNα: interferon-α; TNF: tumor necrosis factor. Arrows define the sequence of processes leading to the establishment of brain metastases.

Extravasation of cancer cells across the BBB may occur via the transcellular or the paracellular route, the paracellular route seeming to be preferred. The transmigration of the BBB by cancer cells requires cell–cell and cell–matrix interactions that modulate BBB architecture and permeability. These interactions involve degradation of extracellular matrix and junctional adhesion molecules, e.g., by lysosomal exocytosis of the cystein protease cathepsin S and signaling via, e.g., the chemokine stromal cell derived factor-1 (SDF1, and CXCL12) and its receptor CXCR4 (for review see [2,65]). It can be assumed that during extravasation of cancer cells across the BBB, processes like exocytosis of proteases, local volume changes during formation of cell protrusions and retraction of the cell rear as well as chemokine-triggered signal transduction pathways rely on Ca^2+^- and electrosignaling which directly depend on ion channel function. The nature of these channels in both, the extravasating cancer cell and the BBB compartment are not defined. The only data in this context on BBB transmigration came from the observation that cytokine-induced downregulation of endothelial tandem pore weakly inward rectifying K^+^ channel (TWIK)-related potassium channel-1 (TREK1, encoded by *KCNK2*) increases leukocyte transmigration through the BBB. Mechanistically, TWIK activity has been shown to downregulate adhesion molecules ICAM1, VCAM1 and PECAM1 and consequently leukocyte–endothelial cell binding [66] (Figure 2, violet).

## 5. Formation of the Metastatic Niche, Neuro-Mimicry, Brain Colonization, and Angiogenesis

Survival of extravasated melanoma and lung cancer cells in the brain parenchyma requires their close physical interaction with the abluminal surface of the blood vessels as demonstrated by real time in vivo imaging of individual cancer cells. This so called co-option of cerebral microvessels by extravasated cancer cells is maintained during dormancy, migration and invasion, a well as proliferation to micrometastases [67]. In the above mentioned study, lung cancer-derived brain metastases expressed vascular endothelial growth factor A (VEGF-A) and inhibition of VEGF-A by bevacizumab prevented angiogenesis and induced dormancy of the micrometastasis [67] suggesting that VEGF-A is a key signaling molecule in early formation of the perivascular niche of metastatic lung cancer cells.

The K^+^ channel EAG1 (K_v_10.1) is expressed in many tumor entities including lung and breast cancer and was found in 5 out of 6, 12 out of 14, and 30 out of 37 brain metastases specimens originated from melanoma, breast, and lung cancer, respectively [65]. Notably, K_v_10.1 has been shown besides stimulating proliferation and migration to boost hypoxia inducible factor-1α (HIF1α)-stimulated upregulation of VEGF-A [68]. Retrospective analysis of survival of patients with brain metastases of different primary tumors treated with surgery and adjuvant whole brain radiotherapy might suggest that patient survival is reciprocally associated with K_v_10.1 abundance in the brain metastasis [65] (Figure 2, turquoise and green).

Beyond the interaction with endothelial cells, extravasated cancer cells have to defend the attack of neural stem cells, microglia and activated astrocytes in their new brain microenvironment. In addition, they have to adapt the metastatic niche to their needs by reprogramming microglia and astrocytes which is facilitated by the pre-expression and up-regulation of a neuronal phenotype by the metastatic cells, a process referred to as neuro-mimicry (for review see [2]). Neuro-mimicry involves overexpression of the above described K_v_10.1 K^+^ channel since the expression of K_v_10.1 in normal tissue is restricted to the central nervous system where it regulates neural excitability [69].

A second neuronal gene upregulated in breast and lung cancer cells with brain tropism is protocadherin-7 (PCDH7). At the metastasis-astrocyte interface, PCDH7 induces the formation of gap junctions between both cell types via assembly of connexin 43 (Cx43) proteins by homophilic interaction with PCDH7 in the astrocyte plasma membrane. Reprogramming of astrocytes occurs by the transfer of the cancer cell-derived second messenger cyclic guanosine monophosphate–adenosine monophosphate (cGAMP) through Cx43 channels. In the astrocytes, cGAMP stimulates the formation and secretion of inflammatory cytokines such as interferon-α (IFNα) and tumor necrosis factor (TNF). Paracrine IFNα- and TNF-stimulated signaling, in turn, supports metastasis growth and chemoresistance [5] (Figure 2, turquoise).

Neuro-mimicry also involves upregulation of ligand-triggered ion channels. Among those are the ionotropic glutamate receptor AMPA type subunit 2 (GRIA2) and the γ-aminobutyric acid receptor type A (GABA_A_R) in brain metastases of melanoma [70] and breast cancer [71], respectively. Glutamate signaling via the Ca^2+^-permeable AMPA receptors has been shown to activate Ca^2+^ effector proteins such as the calcium/calmodulin-dependent protein kinase II (CaMKII) that promotes survival of melanoma brain metastastatic cells. Remarkably, a glutamergic Ca^2+^ overflow and subsequent triggering of cancer cell apoptosis is prevented by gap junction-mediated siphoning of Ca^2+^ into astrocytes [70]. The role of GABA_A_R upregulation in brain metastasis of breast cancer, by contrast, is not defined. In addition to GABA_A_R, breast cancer-derived brain metastases upregulate GABA transporter and GABA transaminase that enable the cell to metabolize GABA as an energy source [71] (Figure 2, turquoise).

Once a macro-metastasis is established, the outgrowing cancer increasingly builds up its own microenvironment and tumor cell survival decreasingly relies on the mimicking of a neuronal phenotype [70]. Rather, metastasis development depends on angiogenesis induced by tumor-derived angiogenetic factors and vasculogenesis mediated by bone marrow derived myeloid cells recruited into the metastasis (for review see [2]). Although the blood–brain–tumor barrier (BBTB) exhibits different morphology and less tightness than the intact BBB, it still inhibits the delivery of most chemotherapeutics to the tumor (for review see [72]). Importantly, activation of Ca^2+^-activated BK_Ca_ K^+^ channels (encoded by the *KCNMA1* gene) [73,74] and ATP-sensitive Kir6.2 (encoded by the *KCNJ11* gene) K^+^ channels [75] in brain endothelial and brain tumor cells has been demonstrated in animal models to increase the permeability of the BBTB. Therefore, concomitant administration of K^+^ channel opener might be a promising strategy to enhance the drug delivery into brain metastasis of patients (Figure 2, green and red).

## 6. Radiotherapy of Brain Metastases

Radiosurgery stereotactically applying high single radiation doses is used to eradicate brain metastasis as an alternative to surgical resection. While the local control of a brain metastasis by radiosurgery is high, it can not prevent the frequent intracranial relapse by the formation of distant brain metastases [76]. Whole brain irradiation is used in the prophylactic setting to lower the risk of developing a brain metastasis [77]. In the therapeutic palliative setting, whole brain irradiation is applied to confine the symptoms of brain metastases such as edema, epilepsy, or neurological malfunctions and delay tumor progression and death. In contrast to radiosurgery, the applicable dose for whole brain irradiation is limited by its side effects on cognitive brain function and does usually not reach doses required to eradicate the metastases.

Ion channels in the plasma and inner mitochondrial membrane have been reported in primary tumors to contribute to the stress response and survival of irradiated tumor cells. In particular, members of the transient receptor potential family of nonselective cation channels such as TRPV6 [78] and TRPM2 [79] or K^+^ channels including K_v_3.4 [80], K_Ca_3.1 (IK_Ca_) [79,81,82,83,84,85], and BK_Ca_ [86,87] are activated in the plasma membrane by ionizing radiation. Mechanistically, these radiogenic channel activities generate Ca^2+^ signals which regulate downstream targets via Ca^2+^-regulated effector proteins such as CaMKII kinases that subsequently interfere with cell motility, cell cycle control or DNA repair [80,82,85]. Uncoupling protein-3 (UCP-3 [88]) and mKATP K^+^ channels [89] in the inner mitochondrial membrane have been shown to confer radioresistance by interfering with reactive oxygen species (ROS) signaling.

Ion channel functions in the cellular stress response of irradiated brain metastasis cells—to the best of our knowledge—have not been studied yet. Many of the above described data, however, were acquired in glioblastoma cell models [82,85,86,87,89]. Importantly, molecular subgroups of glioblastoma resemble brain metastasis cells in many features. Among those are inhabitation of perivascular niches [90], co-option of and migration along vessels [91], expression of neuron markers such as GABA_A_R [92], expression of radiosensitive BK_Ca_ channels [74,91], up-regulated SDF1/CXCR4 signaling [2,90], and gap junction-mediated formation of functional syncytia with astrocytes [93]. Therefore, it might be allowed to speculate, that ion channels contribute to the survival of irradiated brain metastasis cells in a similar manner as observed in glioblastoma cells. If so, deciphering of the involved channel types, their radiation-induced modifications and their functional significance for the survival of the irradiated brain metastasis cells might unveil new strategies to radiosensitize brain metastases.

## 7. Targeting of Brain Metastasis-Promoting Ion Channels

Drugs directly targeting ion channels or inhibiting ion channels by off-target effects are in clinical use or have been tested in clinical trials. The classical neuroleptics haloperidol or chlorpromazine, for instance, block BK_Ca_ K^+^ channels with an IC_50_ in the low micromolar range [94] and accumulate in the human brain to therapeutically effective concentrations [95,96]. Senicapoc (ICA-17043) an oral drug which inhibits K_Ca_3.1 (IK_Ca_) channels with a low nanomolar IC_50_ has been shown to be safe in clinical trials [97] and accumulate to plasma concentrations sufficient to block 70% of K_Ca_3.1 channel activity [17].

A large number of preclinical mostly in vitro studies suggests an anti-tumoral and anti-metastatic action of VGSC inhibitors (for review see [98]). Class I antiarrhythmic drugs and the anticonvulsants phenytoin, lamotrigine, carbamazepine, and valproate are such VGSC inhibitors. A cohort study based on primary care data from the UK QResearch database analyzed among others the overall survival of 2230 breast cancer patients exposed to VGSC inhibitors in comparison with a control cohort of 41,342 breast cancer patients that did not receive VGSC-targeting medication [99]. Unexpectedly, drug exposure was associated with a decrease of overall survival of the breast cancer patients (HR of 1.64 (95% CI 1.51–1.77, *p* < 0.001)). Despite the high patient numbers, interpretation of this study is largely limited because information on potentially important confounders such as cancer stage, co-morbidities and cause of death are not available [99].

Tricyclic antidepressants are K_v_10.1 (EAG1) blockers. In a retrospective analysis of survival of patients with brain metastases of different carcinomas treated with surgery and adjuvant whole brain fractionated radiotherapy and in part with tricyclic antidepressants the effect of K_v_10.1 inhibition on overall survival was addressed. As a result, exposure to tricyclic antidepressants extended in brain metastases patients with low K_v_10.1 protein abundance (but not in patients with high metastatic K_v_10.1 expression) the median overall survival from 10 to 13 months (*p* = 0.03 in univariate testing) [65]. Again, the interpretation of this study is limited due to low number of patients, missing subgroup analysis concerning primary tumor entity, or size, location and number of brain metastases, or degree of surgical resection, applied chemotherapy, etc. Nonetheless, these studies clearly illustrate that subgroups of cancer patients receive ion channel-inhibiting drugs in daily clinical routine due to co-morbidities.

## 8. Concluding Remarks

The role of ion channels in brain metastasis is largely unexplored. The few data available suggest a function of ion channels in epithelial–mesenchymal transition and evasion of cancer cells from the primary tumor, in survival of circulating cancer cells within the circulation, in the mutual adaptation of metastatic cancer cells and their microenvironment in the brain metastatic niche, and in the regulation of the blood–brain–tumor barrier. Some channels such as the neonatal splice variant of Na_v_1.5 (nNa_v_1.5) in, e.g., primary breast cancer cells, or mutated PANX1 (PANX1^1−98^) in circulating cancer cells, seem to be highly cancer cell-associated and might be used to specifically target cancer cells in the future. CLCA2 and ANO1 may be used already today as markers for circulating cancer cells to monitor efficacy of the therapy directed against the primary tumor or to estimate the systemic cancer cell burden. Moreover, animal experiments suggest that co-administration of K^+^ channel activators might be used in the clinic to increase the permeability of the blood–brain–tumor barrier in order to improve delivery of chemotherapeutics to the brain metastasis. Up to now, stressable clinical data on the effect of ion channel-inhibiting drugs on prevention or treatment of brain metastases are missing. Final answers whether or not, e.g., anticonvulsants may prevent brain metasatasis in breast cancer patients or antidepressants may increase the efficacy of anti-brain metatasis therapy can only be acquired by conducting prospective randomized placebo-controlled clinical trials. Those, however, have to await further and stronger experimental evidence for a functional significance of ion channels in brain metastasis, which has to be gained by adequate animal models.
